# ‘Bridging the gap’: exploring shared decision-making with autistic young people within an NHS Learning Disability and Autism Keyworker Programme in England

**DOI:** 10.1186/s12913-026-14025-z

**Published:** 2026-02-02

**Authors:** Emily Ellington, Sarah Parsons, Hanna Kovshoff

**Affiliations:** 1 School of Psychology, Faculty of Environmental and Life Sciences, University of Southampton, Building 44, Highfield Campus, Southampton, SO17 1BJ UK; 2 The Autism Community Research Network @Southampton [ACoRNS], Southampton, England; 3 Southampton Education School, University of Southampton, Southampton, England

**Keywords:** Autism, Shared decision-making, Person centred-care, Dialogue

## Abstract

**Background:**

While each young person has the right to participate in shared decision-making about their care and support, autistic young people often report poor experiences of mental health services and are frequently excluded from, or misunderstood within, care planning conversations. Given that shared decision-making requires practitioners and service users to discuss options together, the differences in communication profiles across autistic and non-autistic populations raises questions as to how mutual understanding can be maximised within care planning conversations. This study sought to explore how shared decision-making took place with autistic young people within a specialist NHS autism community mental health service in England, and to elucidate the features of practice that enabled the effective participation of autistic young people in decision-making discussions.

**Methods:**

A qualitative case study was undertaken within an NHS Autism and Learning Disability Keyworker Programme in England involving 13 participants (4 autistic young people, 3 parent/carers, and 6 service keyworkers). Data were collected through regular service observations, interviews and focus groups across six months.

**Results:**

Four themes were generated to explain how shared decision-making was enacted within and across the service: (1) navigating organisational tensions, (2) bridging different communication styles, (3) enabling autistic thinking patterns and (4) creating conditions for reciprocity and dialogue. Eight subcategories were developed to illustrate distinctive features of practice which enabled autistic young people’s participation in decision-making conversations.

**Conclusion:**

The findings demonstrate that specialist keyworkers were central to facilitating shared decision-making with autistic young people. Their strong understanding of autism enabled them to bridge communication differences across multiple stakeholders and by adapting communication approaches to suit an autistic profile, their practice encouraged meaningful dialogue. The study offers depth and clarity on strategies used by the keyworkers to enable shared decision-making conversations to take place effectively with autistic young people and has wider applicability across healthcare services.

**Supplementary Information:**

The online version contains supplementary material available at 10.1186/s12913-026-14025-z.

## Background

### A right to be involved: shared decision-making

Every person has the right to be actively involved in decisions regarding the care and support they receive from healthcare services in England. This right is enshrined in the Health and Social Care Act (2012) [1] and reflected within the National Health Service (NHS) constitution [2]. It extends to children and young people, whose right to express their views on all matters affecting them is also mandated within Article 12 of the United National Convention on the Rights of the Child [3]. In recognition of these rights, an increased focus on involving all patients in care planning has been driven by the NHS Long Term Plan (2019) [4] which states that “people will get more control over their own health, and more personalised care when they need it” (p.12) and that shared decision-making should be “mainstreamed in primary care” (p. 6).

Shared decision-making is viewed as a cornerstone of person-centred care globally [5] and involves practitioners and patients working in partnership to agree a course of action [6]. It has been described as a desired “middle ground” [5, p.210] between traditional paternalistic models of care (where professional opinion dictates what is best for the patient) and informed-choice models (where the patient is provided with information and trusted to decide independently) [7]. In practice, shared decision-making requires both the professional and the patient to play an active role in discussing care options and in determining a course of action. Within this, all knowledge should be valued to negotiate decision-making through mutual respect and understanding [6]. Using shared decision-making within healthcare services has been shown to improve feelings of autonomy in patients [8] and has also been associated with increased trust in healthcare professionals [9].

Concerns about shared decision-making with children and young people has received specific attention due to questions about their capacity to understand or make decisions about their own care [10]. Nonetheless, studies have shown that involving children in decisions about the care they receive enhances their general wellbeing [11] while excluding them from these discussions can increase their anxiety [12]. In practice, the extent to which children can make decisions about the care and treatment they receive must be determined based on age and/or a developmentally appropriate understanding of the issues at hand [13]. Paediatric care in England therefore often refers to ‘Gillick competence’: an indication that a child has the capacity to consent to their own treatment [14]. Nevertheless, Gabe et al. (2005) stressed that it often “takes three to tango” [10] when it comes to shared decision-making with children and young people, as parent/carers continue to play a central role in supporting, facilitating, and influencing decision-making with children and young people even after they turn 16^1^.

### Considering an autistic profile within young people’s shared decision-making

Enacting shared decision-making with autistic^2^ children and young people has additional considerations, as autistic people process information, experience the world, and communicate differently to non-autistic people. Autistic communication is often characterised by different speech patterns, including the use of direct and literal language [18], longer conversational turns [19] and in-depth focus on topics of interest [20]. Autistic people also rely much less on body language and tone of voice as communicative cues than non-autistic people [21]. At times, these differences in communication styles mean that autistic and non-autistic people can struggle to understand each other [22]. While deficit-led accounts of autism have located the cause of communication breakdowns within the autistic person exclusively [23], recent research has challenged this view. Specifically, the ‘double empathy problem’ [24] suggests that unsuccessful interaction between autistic and non-autistic people is the result of *mutual misunderstandings* when individuals with different communication styles interact. Hence it is a *shared* difficulty with understanding *each other* that results in communication breakdowns. This is supported by research which has shown that it is the neurotype *matching* (i.e. autistic and autistic, or non-autistic and non-autistic) which predicts communication success, not the neurotype itself [25].

Additionally, research suggests that autistic people tend to process decisions differently to non-autistic people. When making a decision, non-autistic people often rely primarily on intuition [26], while autistic people tend to rely more heavily on concrete information [27]. Hence autistic people have been said to possess a more rational and deliberate decision-making style [28]. However, autistic people may also require more time than their non-autistic counterparts to make a decision [29] and having their thinking patterns interrupted can inhibit their decision-making capacity [30]. Additionally, certain decision-making scenarios can cause autistic people significant anxiety, particularly if the decision must be made quickly [31], or within a social circumstance [29].

The communication differences and cognitive processing styles of autistic people have significant implications for shared decision-making practice. Several studies have found that communication difficulties frequently exist within autistic patient-clinician interactions [32] and clinicians have reported finding it hard to build a rapport with autistic patients [33]. Deficit-oriented perspectives have suggested that these difficulties are inherently connected to an autistic profile, with some researchers even cautioning that the “language and social skill deficits associated with ASD could impede the formation of therapeutic relationships” [34, p.54] and that autistic “impairments in social communication can lead to difficulty expressing symptoms” [35]. Yet despite research suggesting that autistic people themselves frequently feel misunderstood within healthcare consultations [36–38], only recently have studies begun to explore how *clinicians* may be contributing to shared misunderstandings, or how they could adapt their practice to better meet the needs of an autistic population [39].

### Positioning shared decision-making as a dialogic practice

As shared decision-making requires deliberation between patients and professionals [40], we suggest it can be considered a *dialogic* practice. By this, we mean a collaborative and relational form of communication where meaning is generated *between* people as they “create something new together” [41, p.3]. This is distinct from a traditional medical model of care which uses a linear transmission model of communication [42]: where a ‘sender’ (the clinician) intentionally conveys a one-way message to a ‘receiver’ (the patient), with no feedback or response (Table 1).

**Table 1:** Comparing features of a traditional model of medical decision-making with a dialogic approach to shared decision-making

	Traditional medical model (linear transmission)	Shared decision-making (dialogic practice)
Type of communication	One-way Clinician → Patient	Two-way Clinician $\leftrightarrows$ Patient
Participant status	*Professional*: expert *Patient*: unqualified	*Professional*: professional insight *Patient*: expert by experience
Knowledge form	Existing knowledge used and perpetuated	New knowledge generated together
Language and terminology	Scientific language dominates and imposes structure over communication	Linguistic presentation of the patient enabled and adopted where appropriate
Clinician mindset	Closed view – no change required	Open approach – accepting and welcoming of change

Understanding the nature of dialogue is central to exploring how shared decision-making unfolds between professionals and autistic young people. To ground this exploration, this study draws on the work of linguist Mikhail Bakhtin (1895–1975) whose conceptualisations of language and communication offer a powerful lens through which to examine the dynamics of voice, power and meaning making in communication. Bakhtin’s theory is particularly relevant to this research because it moves beyond viewing dialogue as a simple exchange of information. Instead, it views this form of communication as a fundamentally reciprocal act and suggests that the contribution of multiple, equally valid, voices (known as *polyphony*) can reshape the way meaning is generated across a society [43]. Within the context of shared decision-making, this suggests that *dialogue* between patients and professionals can create a unique evidence-base by combining experiential and professional perspectives [44]. This may be especially important when working with autistic young people, as involving them in dialogue may help to generate a new form of knowledge that could positively reshape the perspectives of non-autistic professionals. Additionally, the notion of Bakhtin’s *polyphony* [43] allows us to interrogate not only which voices are physically present but also which are heard, validated or marginalised. This is particularly pertinent to autistic people, who often face additional barriers to being understood and taken seriously in professional discourse [37].

Despite its equalising potential, Bakhtin also believed that dialogue could be impeded by power imbalances and social hierarchies [43], concepts commonly cited as barriers to shared decision-making practice [6, 7]. To illustrate this, he proposed that two opposing forces act on the construction of language. A *centripetal* force pulls individuals *towards* social conformity and a unified presentation of speech and language. Examples of this can be seen in the use of organisational jargon which creates a perception of professional expertise [45]. Yet simultaneously, a *centrifugal* force pushes individuals *away* from conformity, towards their own unique linguistic presentation. This can be seen in the use of slang and colloquialisms, for example. Within the context of healthcare services, these opposing forces can be seen within patient-professional discourse as the use of different language and terminology on each side of the communicative exchange may create tension between parties [46]. Indeed, following analysis of interviews with 77 executives from across an NHS Trust, Lord and Gale (2014) [47] argued that while commitments to person centred practice were made, the “objective processes” driven by executives dominated the “subjective experience” [42, p.726] of patients, devaluing their voice and limiting their participation.

These opposing linguistic styles could also explain why autistic people have been found to experience *greater* difficulty than non-autistic patients when engaging with healthcare professionals [38]. Not only can tension be created between the different terminologies used by professionals and patients, but strain may also be felt between the different semantic styles used by non-autistic and autistic people. This has been described as a ‘triple empathy problem’ by Shaw et al. (2023) [48] who suggested that the combination of differences in professional versus lay terminologies, and autistic versus non autistic viewpoints, means that “it proves even harder for autistic patients to see their (non-autistic) doctor’s perspective, and even harder for (non-autistic) doctors to see autistic patients’ perspectives” [43, p.1754].

However, despite the clear differences in communication, interaction and cognitive processing that autistic people experience, there is a paucity of research exploring shared decision-making with autistic young people specifically. Where it does exist, almost all research has focused heavily on the views and experiences of parents and carers (11; 49–51). Privileging the views of parent/carers, and using parents as proxies for autistic children’s experiences, has been heavily criticised by researchers who suggest that autistic children’s voices are “scarcely heard” [47, p. 3]. Mullally et al. (2024) [52] further argued that for autism research to become more comprehensive, *all* autistic voices must be “placed front and centre stage” [47, p.3]. This study therefore aimed to focus on the experiences of autistic young people themselves within shared decision-making and to explore how a specialist healthcare team enacted shared decision-making with this cohort. Specifically, we wanted to address the following research question: what features of practice with a Learning Disability and Autism Keyworking Programme enabled or inhibited autistic young people’s participation in shared decision-making conversations?

## Methods

### Study design

This qualitative case study was undertaken within an NHS Learning Disability and Autism Keyworking Programme (LDAP) in England. Data were collected in sequential phases using three methods: first participant observation was undertaken, then semi-structured interviews were conducted with young people and parent/carers, and finally a focus group was held with the LDAP keyworkers. In total, over 40 h of participant observation was undertaken over a period of six months from June 2023 – November 2023, 6 semi-structured interviews were conducted, and one focus group was held lasting 1.5 h.

### Study context

The study was conceived in partnership with the LDAP, following an approach from a senior manager within the service to the Autism Community Research Network @ Southampton (ACoRNS; a research-practice partnership based at the University of Southampton) [53]. The service had expressed a desire to participate in research that would help the service to better meet the needs of autistic young people. Following this, the lead author (a PhD student under the supervision of the second and third authors) established a relationship with the service and the focus of the investigation was determined in partnership with the service manager following several months of collaborative discussions.

### The case

The case is a LDAP within a single NHS trust. It was established in 2022 as part of a national NHS pilot to reduce inpatient numbers across the autistic population. The service works with children and young people (CYP) up to the age of 25, who are autistic and/or have a learning disability and may be at risk of hospital admission due to mental ill-health. It provides a short-medium term intervention lasting up to 6 months through which a specialist keyworker will work in partnership with the CYP, and their family, to co-produce and implement a sustainable person-centred plan to prevent exacerbation of symptoms that would lead to hospital admission. Once the risk of hospital admission is reduced, the allocated keyworker will step back from the case. The keyworkers’ day-to-day activities vary dramatically depending on the life circumstances and needs of the young person and family they are supporting and include: attending professional meetings, liaising with other professionals on case progress/coordination, identifying community activities for the young person to participate in, supporting a young person with attendance at educational or community-based activities, developing communication passports, helping to prepare CYP for transition, and visiting young people within secure and/or hospital placements. A key feature of the keyworker’s role is to ensure that autistic young people’s voices are heard, understood, and respected within care planning [54].

### Participants

Three participant groups were purposively recruited from within the case: (1) autistic young people under the care of the service, (2) parent/carers of young people under the service and (3) keyworkers working within the service. Information about the study was shared with all young people known to the service and their families via email and hard copies were provided by keyworkers during home visits. An accessible version of the study information was provided alongside the original version and notification of the study was also placed in the service newsletter.

In total, 13 participants were recruited (4 = young people, 3 = parent/carers, 6 = keyworkers). The young people recruited were all aged 17–19: two were young women and were two young men. All four had a diagnosis of autism and were verbal communicators without learning disabilities. Two of the parent/carers were mothers of the participants, while one was a grandmother. Six young people and families understandably declined to participate as they advised that they were too close to crisis. Each young person and parent/carer participant were compensated with a £25 Amazon voucher as a token of gratitude for their participation.

### Ethics

Ethical concerns have been raised around conducting research with participants who are deemed ‘vulnerable’. These include concerns about certain populations being at greater risk of manipulation and harm [55]. Nevertheless, we agree firmly with Drewett and O’Reilly (2023) [56] who argued:

“Autistic people and inpatients are still frequently excluded from research based on their perceived ‘vulnerability’, the complexity of accessing them and concerns about their capacity to provide informed consent. While it is essential to safeguard this group from exploitation, it is equally important to ensure that they are not denied opportunities simply because of their autistic or hospital status” [51, p.827].

We took several steps to try and support autistic young people known to the LDAP to be involved in the study whilst also ensuring fully informed consent and reinforcing their right to withdraw at any time. Firstly, an accessible version of the study information was provided to young people to support their understanding. Additionally, young people were given the opportunity to discuss the study with their keyworker: someone they already had a trusting relationship with. Written informed consent was obtained from each participant upon the commencement of the study and consent was reconfirmed verbally at the outset of each field observation and in advance of interviews being undertaken. Throughout all data collection, the researcher remained alert to any non-verbal signs that may suggest a participant was uncomfortable or distressed, although this did not occur. As all four young people recruited for participation were aged over 16, informed consent was sought and secured from them directly. Ethical approval was secured for the study from University of Southampton’s School of Psychology ethics review committee (Ref: 80104).

## Data collection

All data collection for the study was undertaken by the first author and so all reference within this section to ‘the researcher’ relates to them.

### Observations

In total, 16 separate field visits were undertaken amounting to over 40 h of participant observation. The focus of key decisions being made for each participant young person during the study are outlined in Table 2. Before observations commenced, a one-page profile of the researcher was shared with all participants to increase their familiarity with the researcher before she entered the field. Observations were arranged in line with the keyworkers’ working schedules whereby the researcher shadowed keyworker visits to the participant young people.

**Table 2:** Shared decision-making focus during data collection

Participant	Shared decision-making focus	
Young person 1 Age 18 Male	*Participant specific decision-making scenario*: Transition to adult social care and associated package of care/community support	*Service care planning decision-making for all participant young people*: Determining outcomes and direction of service intervention from the LDAP
Young person 2 Age 17 Female	*Participant specific decision-making scenario*: College course choice	
Young person 3 Age 17 Male	*Participant specific decision-making scenario*: Further education/employment choice	
Young person 4 Age 19 Female	*Participant specific decision-making scenario*: Discharge from in-patient facility and community care planning	

During field observations, the researcher was positioned as a participant observer whereby she directly interacted with participants to “elicit narratives” [52, p.2]. This was done to clarify her understanding of what was happening and seek further information on the context and views of the participants. For example, the researcher asked the participants present during the observation if they could: clarify the scope of the decision that was being discussed, explain why they chose to do something, or confirm whether they consciously and/or deliberately used a particular approach within their practice. Observational data was collected through handwritten fieldnotes made in situ by the researcher. These were unstructured but placed a focus on how the decision-making conversations were being enacted, what was happening in the moment, and the circumstances surrounding the social context. Following each field visit, the researcher completed a field record and typed up all field notes into a Microsoft word template on the evening of the observation.

### Semi-structured interviews

Semi-structured interviews were held with three out of four young people who participated in the observation phase. One young person felt unwilling to participate in an interview but consented for their observational data to be included. Interviews were also held with a parent/carer of each of these three young people. A copy of the interview schedule was shared with young people (see Additional Material 1) and parent/carers (see Additional Material 2) in advance to maximise their awareness of what would be asked. Participants were given the option to participate in person or online: two young people chose to be interviewed in person at their home, while one chose for this to be completed online. One parent/carer was interviewed online and two were interviewed in person.

Interview questions focussed on participants’ experiences of shared decision-making within the service and their views on factors that enabled or undermined this for them or their child. Scenarios that had been observed through the observation phase were probed for further clarification or comment from the participants to allow for greater triangulation of fieldnote data. All interviews were audio recorded and transcribed for analysis.

### Focus group

All six keyworkers participated in a focus group once the observation phase and interviews were completed. A copy of the questions posed to the focus group was shared within participants in advance (see Additional Material 3) and the focus group was facilitated online by the researcher. Questions focussed on how they attempted to engage young people in shared decision-making, when they felt this had been successful and what they thought was useful. The focus group was held online and lasted 1 h and 20 min. The session was audio recorded and transcribed for analysis.

### Analysis

Data analysis was led by the first author as part of their PhD research, under the supervision of the co-authors. The first author developed the initial coding frame, which was subsequently reviewed by the co-authors and who provided feedback and suggested revisions. These contributions informed the refinement of the coding frame. Data coding and theme development were primarily conducted by the first author, with ongoing input and critical discussion provided through regular supervisory meetings with the co-authors.

All data were uploaded to NVivo [58] and analysed using hybrid thematic analysis, a method of qualitative analysis that combines inductive and deductive approaches [59]. The deductive part of the analysis involved the development of a coding frame relating to core Bakhtinian concepts of dialogue (see Additional Material 4) while the inductive part sought to generate insights directly from the data that may be more unique to autistic experiences. In its entirety, the analysis involved eight stages, devised by the first author as an adapted version of Fereday & Muir-Cochrane’s (2006) [56] approach (summarised in Table 3). When undertaking full data coding, (Table 3, Stage 5) the deductive coding was undertaken first. Data-led coding using an inductive approach was then completed to ensure the voices and experiences of participants were not obfuscated by the theoretical lens. Codes were then connected and collapsed across deductive and inductive coding to integrate theoretical and data-led concepts.

**Table 3:** Stages of analysis

1	Developing the deductive coding frame
2	Reviewing and refining the coding frame (1)
3	Test coding
4	Reviewing and refining the coding frame (2)
5	Full data coding
6	Connecting and collapsing codes
7	Developing categories
8	Developing themes

To develop the final themes, multiple analytic techniques were used to strengthen interpretive consistency. Firstly, critical analytic memoing [60] was undertaken, where the researcher wrote out all categories and annotated the document with thoughts, arrows, and tentative connecting lines. Secondly, one mode network modelling [61] was used to help identify connections between individual categories. Connections were then mapped visually into a network model [61] to guide analysis towards the development of themes. Ultimately, themes were developed by reviewing all analytic stages with particular focus on what was happening across connected categories. This was heavily centred around what Lipton (2017) [62] describes as trying to make an “inference to the best explanation” [57, p.184] and involved integration of both deductive and inductive insights.

## Results

**Fig. 1:**
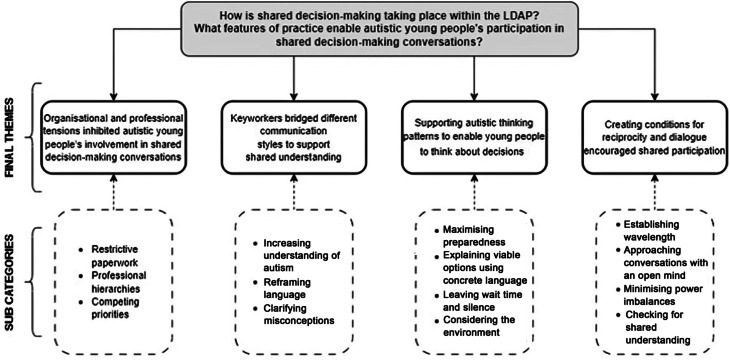
Overview of study findings

Four themes were developed in response to the research question, underpinned by 14 subcategories. An overview of the findings is provided in Fig. 1.

### Theme 1: Organisational and professional tensions inhibited autistic young people’s involvement in shared decision-making conversations

Theme 1 captures organisational and professional tensions that existed and illustrates how these inhibited young people’s involvement in shared decision-making conversations. The theme was generated from three subcategories: (1) *restrictive paperwork*, (2) *professional hierarchies*, and (3) *competing priorities*. These are discussed in turn.

#### Restrictive paperwork

Standardised ‘Family Plans’ were used across the LDAP to guide decision-making and to direct the service intervention, but the paperwork was highly restrictive. While keyworkers made attempts to co-develop the plans with young people, the format of the paperwork seemed to inhibit meaningful conversations. The plan template was extremely formulaic and used advanced language which meant that young people often struggled to understand its content or purpose. When keyworkers tried to follow the format of the plans during meetings with the young people, it appeared that conversations were stunted, and young people lost interest and focus. Keyworkers highlighted the tension they felt between needing to complete these plans for administrative purposes while recognising that their format was not “autism-friendly” (Fieldnote).

#### Competing priorities

In addition, professionals beyond the LDAP seemed more focused on their own organisational priorities than on meaningfully including the young people in shared decision-making. The keyworkers often reported that professionals working outside of the LDAP viewed decisions that were ‘required’ for their service as the greatest priority but that this detracted from the importance of facilitating meaningful discussions with young people. This was also observed during the observation phase. For example, during a multi-professional meeting held to discuss the next steps for an adult social care plan, there was little attempt made by a social worker to explore the available options fully with the young person and their family:

Social Worker advised she needed to get this [the care plan] completed and so was updating the plan during the meeting. She narrated actions and then sought confirmation from Mum/YP that they agreed. Following the meeting, the keyworker advised that she felt uncomfortable as there was a lack of information provided in relation to what was happening/what the options were for the young person (Fieldnote).

Similarly, in another case, an educational placement had progressed a decision relating to a young person’s choice of course without providing sufficient information about the qualification implications of the course choice. From the keyworker’s perspective, this was done as the “college wanted to ‘get enrolment done’ and deal with the consequences afterward” (Fieldnote). They also explained that this negatively impacted the young person who was left “struggling to process what’s going to happen, as he is fixed on the previous advice and cannot understand why this has now changed” (Fieldnote). Hence the competing priorities of other professionals often seemed to take precedence over engaging young people in meaningful discussions, reducing their involvement and participation in shared decision-making.

#### Professional hierarchies

Furthermore, professional hierarchies sometimes impacted the way young people were involved in decision-making conversations. This was evident in the way some professionals outside of the LDAP spoke to young people and also how they physically positioned themselves during discussions. “They will walk in and stand there with their arms folded. There’s no, getting down to her level. There’s no soft speaking” (P/C 2 Interview). This was illustrated by one young person who explained, “See, that’s the thing. “Professionals”, that’s the way they separate you. They’re like oh we’re professionals you’re not. It’s like, we’re still people. Just because you’re a professional doesn’t mean you’re worth anymore or know any better*”* (YP 1 Interview). Consequently, young people did not feel as though conversations with all professionals were held on an equal footing which inhibited their participation. Nonetheless, keyworkers seemed to be aware of this and actively tried to equalise and redistribute power between themselves, young people and their families. This is discussed further in Theme 4.

Keyworkers also experienced difficulties with professional hierarchies. They sometimes experienced negative power dynamics within cross-service discussions and shared that they felt as though they were perceived to have a “lower status” (Keyworker Focus Group) by some professionals. Consequently, keyworkers felt that some other professionals were less willing to work in partnership with them to maximise the young person’s involvement. This was also noted by a parent/carer who explained:

I think the KW role needs to be much more senior. They are underselling them. If people think oh, you’re not senior enough, you don’t really know what you’re talking about. And so that has an impact on [name of child] because people aren’t listening to the people they should be listening to (P/C 2 Interview).

### Theme 2: Keyworkers bridged different communication styles to support shared understanding

Theme 2 illustrates how keyworkers played a critical role in enabling shared understanding between autistic young people, parent/carers and professionals. It is explored through three connected subcategories (1) *increasing understanding of autism* (2) *reframing language* and (3) *clarifying misconceptions.*

#### Increasing understanding of autism

Keyworkers recognised that autistic young people and non-autistic professionals communicate, interact and process information differently: “We have to think about how that autistic young person processes information and actually make sure that they understand what we are saying, or what the options are in response to a question, because often that’s very different from other people” (Keyworker Focus Group). Consequently, they played an active role in trying to make sure that all professionals better understood an autistic profile so that shared decision-making discourse could happen effectively. This was underpinned by a strong knowledge and understanding of autism, and a recognition that communicative adaptation was required to maximise autistic young people’s participation. This was noted by the young people and parent/carers who felt the keyworkers placed an “autistic lens” on professional discussions which supported shared understanding: “I think if we didn’t have [name of keyworker] and her knowledge of autism, and her constantly using the autistic lens, they just wouldn’t have understood [name of child] and she wouldn’t have understood them” (P/C 2 Interview).

#### Reframing language

Keyworkers also reframed language that may have been misinterpreted or misunderstood on both sides of interactional exchanges. This is exemplified by a keyworker who explained the following:

Open questions for our cohort are usually a big no, no. But that goes totally against the training that all of the mental health teams and social workers and everyone are given – they’re told to be as open as possible. So, it goes totally against the grain for them. So, part of the process is actually enabling the professionals to understand why those open questions are so inaccessible for our youngsters, and that can be a battle in itself once, but once they do and you’ve modelled that to them, it can work so much better (Keyworker Focus Group).

Keyworkers therefore often referred to themselves as “translators” or as people who would “bridge the gap” (Keyworker Focus Group) between professionals, parents, and autistic young people. One keyworker described her role in this regard as follows:

I see it as I’m trying to support the young person to make the most of the support that’s there and to actually enable them to be to communicate what they need to communicate. I’m not a clinician, so I, you know, it’s a really true collaboration, and it feels very natural when it works. For example, with the psychiatrist that I’ve been working with up in [name of location], she said I would never have thought to do it this way. She said, I just wouldn’t have got any of that information without you. (Keyworker Focus Group).

Indeed, communicative adaptation led by the keyworkers was remarked on by a young person who suggested that his keyworker “changed that way she kind of talked, she basically changed a lot to kind of fit my needs” (YP 2 Interview) and that she helped other professionals to do this too. “It seems like everyone does it more that way now. Which is good” (YP 2 Interview) A parent/carer also explained how a keyworker helped them to adapt their own conversational style when discussing things with their child: “I think she did that for me as well. She showed me how to talk to him when discussing these things” (P/C 2 Interview).

#### Clarifying misconceptions

Keyworkers also clarified misconceptions that may have inhibited shared decision-making. For example, they were able to identify when information may have been misunderstood by young people and took steps to ensure that the information was revisited in a different way. Identifying these misconceptions was rooted in a deep understanding of autism but also in a strong knowledge of the individual young person’s profile and presentation:

I can see those moments where professionals think they’ve communicated something and because that youngster has nodded, they don’t see that they’ve gone into their shell, they’re just blank. They haven’t understood. I can notice that because I know them and I make sure we revisit it (Keyworker Focus Group).

Importantly, misconceptions appeared to occur on both sides of the interactional exchange and keyworkers also remarked on how frequently professionals would misinterpret young people or hold mistaken beliefs about what they wanted. This is exemplified by the following example:

I have a young lady that I’m working with who’s communicated numerous times to say that she wants to go home, and all professionals across the network are all jumping on that. Fantastic, right? But she doesn’t. When she says home, she doesn’t mean I physically want to go home. So, it’s always a question of, you know, kind of having that insight, staying curious and not just taking those words at face value because this young person in particular is just exceptional at masking. She will say the words that she expects to be the right or welcomed kind of answer but that isn’t actually her saying her piece. She’s not really saying what she wants (Keyworker Focus Group).

This highlights the keyworkers ability to recognise when communication may have broken down, often in subtle and often overlooked ways. Their insight into both autism and the individual young person helped to ensure that misconceptions were addressed so that all stakeholders could understand each other more effectively.

### Theme 3: Keyworkers supported autistic thinking patterns to enable young people to think about decisions

Theme 3 outlines distinct strategies keyworkers used to help autistic young people to think about the decisions they were making. These are discussed within the context of four subcategories (1) *maximising preparedness*, (2) *explaining viable options using concrete language*, (3) *leaving wait time and silence*, and (4) *considering the environment*. These strategies were critical within the context of shared decision-making, as young people had to be supported to *think* about the decision at hand before being able to communicate their position on it. Hence it is important to note that *enabling autistic thinking patterns* worked in tandem with theme 4: *creating conditions for reciprocity and dialogue.*

#### Maximising preparedness

The keyworkers made sure the young people they supported were well-prepared for decision-making conversations. They gave notice of decisions that would be made during visits and were explicit about what would happen during their appointments and what would be discussed. Young people seemed to value the preparation their keyworkers provided and specifically expressed the positive impact that advance preparation had on their decision-making ability: “I like things in advance so I can think about it. Maybe one day or one week” (YP 3 Interview).

However, it was apparent that not all professionals outside of the LDAP took steps to maximise preparation for the young people. For example, in one case, a keyworker was helping a young person to prepare for a meeting to determine their subject options at college. They explicitly notified the college of the need for the young person to be well prepared for any meeting. Despite this, the college called a young person into a meeting with no notice. Consequently, the young person did not feel ready for the meeting to take place and expressed extreme dissatisfaction about the lack of notice they were given and the negative impact it had on their ability to participate in the decision-making discussions.

#### Explaining viable options using concrete language

Similarly, young people preferred structure for the decision-making conversations, by being provided with concrete options rather than simply being asked what they wanted in an open-ended way:

**Young Person**: They usually just ask what I would want. Which I don’t like. Because I don’t like being, I don’t know how to explain it. I don’t like being asked questions with a broad answer, I need like options to pick from I can’t just make up a decision myself like that.

**Reasearcher**: Ah, I see. So, if someone asks you to make a decision, you would prefer if they gave you a set of options to choose from?

**Young Person**: Yeh exactly. I hate it when people just say things like ‘what do you want us to do to help?’ I hate that. (YP 2 Interview).

Keyworkers would therefore outline all viable options explicitly for young people, so there was a structure to the decisions they needed to think about.

#### Leaving wait time and silence

Often, the young people needed time to process what had been said to them during conversations before being expected to respond. Several field notes remarked on the amount of ‘wait time’ keyworkers provided to young people during decision-making conversations, and this was stressed as important by a young person: “I’m like my friend. Sometimes you ask her a question and sometimes it takes her so long to process what you’ve said then it might be like 30 seconds later she’ll give you an answer. You just need to wait” (YP 2 Interview).

#### Considering the environment

Additionally, the environment in which decision-making discussions took place seemed to either enable or inhibit young people’s participation. All participants remarked on finding it hard to process information when struggling with sensory processing. Examples of traffic noise being overwhelming and the sensation of certain fabrics being unbearable were given. Consequently, all young people expressed a preference for having decision-making conversations at home (where they could control the environment) as opposed to in clinics, schools, or other professional offices.

Nevertheless, even during home visits, keyworkers were aware of the impact their physical proximity may have on young people’s sensory processing. Consequently, they frequently checked with young people where they would prefer them to sit and would often re-check whether their position and tone/volume of voice was comfortable for the young person throughout their visits. By paying close attention to these environmental factors, keyworkers created more accessible spaces for conversational engagement.

### Theme 4: Creating conditions for reciprocity and dialogue encouraged shared participation

Them 4 relates to specific conditions created by the keyworkers, rooted in a sense of reciprocity, that seemed to enable meaningful discussions to take place. Those conditions are highlighted by the following four subcategories: (1) *establishing wavelength*, (2) *approaching conversations with an open mind*, (3) *minimising power imbalances* and (4) *checking for shared understanding*.

#### Establishing wavelength

Establishing wavelength refers to an act of attunement that was evident across keyworkers’ practice. This involved the keyworkers taking steps to ‘tune in’ to the young person and establish a sense of reciprocity and rapport. They would explore young people’s interests, share personal anecdotes, and make attempts to arrange visits in locations the young people would feel most comfortable. This was appreciated by the young people and seemed to establish a sense of connection and respect between them. “She takes on my special interests as well, she knows I get obsessive over random stuff, and she tries to base out meetings over that to keep me occupied and interested” (YP 2 Interview).

#### Minimising power imbalances

Keyworkers also appeared to try and equalise power imbalances between themselves, the young people, and their families. They all expressed a conscious attempt to make themselves “unthreatening” (Keyworker Focus Group) to young people and frequently did this by positioning themselves as an ‘unprofessional’ – someone who does not conform to the expected formality of a ‘professional’. This was observed in several different ways. There was a common use of humour by the keyworkers, including poking fun at themselves, and they deliberately dressed and presented in an informal way. Their conduct during visits was relaxed and personal; they would sit on the floor, eat and drink alongside the young people, and join in with games and activities. One young person specifically described their keyworker as “a different kind of professional” (YP 2 Interview).

While this was perceived favourably by the young people and their families, and seemed to help the keyworkers establish a positive rapport with those they were supporting, there were times when their ‘unprofessional’ presentation may have exacerbated the negative power dynamics discussed earlier within the context of *professional hierarchies.* For example, a keyworker explained that “I was in a meeting once, there were 25 regional psychiatrists in this meeting, and I was the only non-psychiatrist. And boy did I know it! I could just sense they looked at me like I wasn’t one of them from the second I walked in like this *indicated to clothing*. Looking at me like, what could I know, you know?” (Keyworker Focus Group).

#### Approaching conversations with an open mind

Relatedly, all keyworkers maintained an open mind when approaching decision-making conversations. This was important to the young people who did not want to be judged by preconceived notions of autism or mental health:

Obviously, they have to read your risks and history – but don’t treat us according to our history. Treat us as a person. Don’t prejudge. Go in with an open mind. Yes, this could happen but don’t expect it to happen (YP 1 Interview).

Keyworkers were willing to be led by the young person’s voice and recognised that young people’s views, perceptions and wishes could change over time. Consequently, they remained responsive, open to change and reinforced to young people that iterative discussion was a positive step within decision-making. “It’s alright, you know, if you said this one day and you say something different the next day, that’s absolutely fine. We are all exploring” (Keyworker, Fieldnote).

#### Checking for shared understanding

Finally, keyworkers constantly checked for shared understanding during conversations. To do this, keyworkers would reflect young people’s contributions back to them and ask the young person to confirm that this was indeed what they meant. Once they had confirmed they had understood a young person correctly, they would often adopt language and terminology used by the young people throughout the rest of the conversation. Keyworkers also sought to check that young people had understood what *they* had said, by asking them to explain what they thought the keyworker meant. These steps highlighted instances of misunderstandings and provided the opportunity for meaning to be clarified between them.

This element of practice was important, as young people were not always able to clearly articulate their thoughts in every conversation. A keyworker explained that this required professionals to “‘listen with your eyes’ as the young people will not always directly tell them what they want/how they feel” (Fieldnote). Additionally, it was noted that young people may not always say what they mean when being asked about decisions. Specifically, young people reported shutting down, or giving any response to end the discussion, when they were feeling overwhelmed. This was also noted by a parent/carer who explained, “if he’s in a meeting and he’s had enough, he will just agree with everything. Although he probably doesn’t actually agree, he will” (P/C 3 Interview).

## Discussion

### Summary of main findings

Calls for shared decision-making are clearly articulated within the NHS national strategy *Universal Personalised Care* [63]. However, this model of practice requires specific investigation with autistic people as they often feel unsupported by traditional practice approaches [64]. This study aimed to understand how the voices and experiences of autistic young people were included in decision making within an LDAP Keyworking Programme, through participant observations, interviews and a focus group discussion. Through a case study approach and multiple data collection methods, it was possible to triangulate the data collected over an extended period of time, and to combine autistic insights with non-autistic professional experiences to generate a rich picture of how shared decision-making was happening with this service and cohort.

The findings highlight factors that enabled and inhibited shared decision-making at an organisational, interactional and individual level. This is in keeping with Waddell et al.’s (2021) [65] systematic review which found that barriers to shared decision-making existed at clinician, patient, organisational and system levels. In our study, two of the organisational barriers appeared unconnected to supporting autistic young people specifically. Rather, the findings relating to *professional hierarchies* and *competing priorities* reflected more general issues which have both been found to exist across a range of different clinical contexts with diverse populations [66].

Standardised paperwork has also been identified as barrier to person-centred care across general healthcare research [67]. But the issues it presented within this study relate to an autistic population specifically. The formatting and language used within the Family Plans was not well understood by the autistic young people and keyworkers unanimously felt it was not “autism friendly”. Their view is supported by research which has found that autistic people find several aspects of standardised paperwork problematic, especially when it includes “the use of difficult vocabulary, confusing terms, or figures of speech, complex sentence structure, confusing grammar, or incomplete phrases” [68, p.61]. Yet while adapting paperwork to better suit the communication needs of autistic people has long been suggested across educational practice [69–71], it receives less attention across healthcare services [72].

At an interactional level, keyworkers were central to enabling autistic young people’s participation in shared decision-making conversations. Previous research has suggested that keyworkers play a vital role as a patient advocate [73] and often improve multi-professional collaboration by helping to “overcome professional culture barriers that result from ineffective team communication” [74, p.469]. However, while Sloper et al. (2006) [75] found that keyworkers for families of disabled children were often used to “speak *on behalf* of the family when dealing with services” (70, p. 156, our emphasis), our findings suggest the LDAP keyworkers enabled young people to participate in shared decision-making *themselves*. Specifically, they ‘translated’ communication across stakeholders and helped other professionals to adapt their communication approaches so that autistic young people and professionals could understand each other better. This contrasts with paternalistic approaches to decision-making whereby professionals speak *for* patients [6] and instead, seemed to increase agency in the young people who were empowered through a sense of shared understanding with professionals.

From a dialogic perspective, these findings suggest that keyworkers were attuned to the different forces shaping language and dialogue. They were able to identify and respond to the centripetal forces pulling professionals towards corporate or medicalised terminology, and also to the centrifugal forces pushing autistic young people towards non-normative language patterns. Recognising and responding to these dual forces has been found to improve shared engagement in other professional contexts. For example, Jaspers (2014) [76] found that a teacher who used stylised language which reflected the linguistic presentation of urban youth (centrifugal force), alongside the standardised language expected by school policy (centripetal force), “kept pupils on board in the classroom through recognizing their backgrounds and valuing their linguistic expertise” (p. 390). Similarly, by ‘translating’ communication between different linguistic styles, keyworkers facilitated effective conversations between autistic young people and professionals, fostering better understanding and collaboration.

Moreover, by recognising the need for non-autistic professionals to adapt their communication approach, the keyworkers’ practice responded directly to Milton’s (2012) [24] double empathy problem. This is particularly significant within a healthcare context where potential mismatches between clinical and lay language, combined with communicative differences across autistic and non-autistic profiles, can lead to a ‘triple empathy problem’ [48]. Hence by acting as ‘translators’, keyworkers were able to mediate autistic/non-autistic communication differences, and also clinical/experiential disjuncts. This could not have been done without the keyworkers’ strong understanding of autism and their self-positioning as an ‘unprofessional’. Importantly, this supports autistic people’s calls for a greater awareness and understanding of autism across the healthcare establishment [77] and aligns with young people’s preferences for informality and authenticity when developing relationships with professionals [78].

The conditions for reciprocity created by the keyworkers were central to enabling young people to participate equally in shared decision-making. By deliberately adopting a stance as an ‘unprofessional’, they helped to level traditional power dynamics and created space for open, non-threatening exchanges to take place. Their use of informality, humour and a strong sense of relatability can be understood through a dialogic lens, particularly in relation to Bakhtin’s concept of the “carnivalesque” [43]. This concept suggests that in moments of carnival, the usual rules of social behaviour are relaxed, and social hierarchies are suspended. Laughter – a key feature of the carnivalesque – acts to degrade power and enable those with less social force to contribute freely to a dialogic space. In the context of this study, the keyworkers use of informality and humour served as a tool to disrupt conventional healthcare hierarchies, empowering autistic young people to express themselves without fear of judgement or dismissal.

More generally, the keyworkers’ approach was highly relational and rooted in a willingness to be open-minded about what may emerge in conversations with autistic young people. Cayer (2005) [79] described this kind of open-mindedness as a “non-judgmental way of listening” (p. 174), highlighting that in order to develop a genuine dialogue where individuals are valued and enabled to participate equally there must be a conscious effort to “talk without agenda” and try to “understand the experience of the other” (p. 175). This approach is consistent with recommendations from Serrano et al. (2016) [80] who emphasised that using conversational dialogue to elicit and explore both patient’s and professional’s views and perspectives is central to good shared decision-making practice.

Our findings also suggest that autistic young people needed to be supported to *think* about a decision in order to engage in meaningful conversations about it. Accordingly, the strategies used by keyworkers to help young people think about the decisions being discussed were supportive of an autistic cognitive processing style [79]. Specifically, their attempts to *maximise preparedness* aligns strongly with findings from Silver and Parsons (2022) [81] who explored the perspectives of autistic adults on strategies that helped or hindered conversations with non-autistic others. They found that “autistic participants reported that they liked to be clear about the conversation topic” (p. 9) at the outset of any discussion and suggested that this reduction in uncertainty about what would be spoken about was helpful to enable autistic participation. Similarly, *leaving wait* time between conversational contributions has also been found to be helpful for autistic people [82] who commonly experience longer processing times than non-autistic people [83]. However, research has also suggested that longer pauses during conversations are often perceived as awkward by non-autistic people [84] who will frequently try to ‘fill’ silences that extend beyond 2 seconds [85]. Nevertheless, providing sufficient wait time for thinking after questions may be especially necessary for autistic people within the context of care planning, as the high speed of communication across healthcare services has been found to negatively impact their ability to process essential information [86].

Providing young people with options using clear and concrete language was another supportive strategy, in keeping with findings which suggest that autistic people can struggle with open ended questions and types of conversation that lack structure such as ‘small talk’ [87]. Within the context of healthcare consultations, Hamdan and Bennet (2024) [86] suggested that “providers should intentionally use clear short sentences, concrete language, direct or prompted questioning, and detailed answers and explanations (p.5)”. This is echoed by Araujo et al. (2022) [88] who found that simplified and concrete language aided autistic young people’s understanding during consultations with healthcare professionals.

Finally, the keyworkers’ practice of *checking for shared understanding* was critical for working with autistic young people as it helped to prevent the risk of mutual misinterpretation that can occur across autistic – non-autistic exchanges. This has also been deemed essential by Haydon et al. (2021) [89], who argued that “it is vital to check for mutual understanding throughout all healthcare encounters” (p. 4) when working with autistic patients. In our study, by reflecting young people’s contributions back to them and by asking young people to clarify what they understood the keyworkers to have said, keyworkers were able to identify areas of misunderstanding and reframe the conversation accordingly. At times, this involved adopting the language used by autistic young people – a strategy also found to be helpful by Silver and Parsons (2022) [81], who suggested autistic participants found it “helpful when their words were accepted, understood, and then used with shared understanding by their communication partner” (p. 10).

### Study limitations

This study highlights practice within one community mental health service in England and as such, the findings are contextually related to this service alone. Additionally, it was not possible to recruit all young people supported by the service to the study as many felt unable to participate. This is a common issue across health care research where access to highly vulnerable participants is regularly cited as a challenge [90]. Consequently, the case is limited by a small sample that does not represent all service users at the time of the study. Similarly, the sample represented within this case comprised of autistic young people who were verbal communicators without co-occurring intellectual disabilities. This could limit the applicability of the findings to populations who use speech to communicate. Nevertheless, the study provides rich insights into the practice of shared decision-making with autistic young people who communicate verbally and offers depth and clarity on strategies which effectively enabled their participation.

### Implications for research and practice

Most importantly, our findings demonstrate that autistic young people *can* be included within shared decision-making but critically, non-autistic adaptation is required to enable their meaningful participation in related conversations. In this case, the use of specialist keyworkers with a strong understanding of autism appeared vital to ensure shared understanding across all stakeholders. Consequently, while mainstream professionals may lack confidence in adapting their practice for an autistic population [91], greater investment in specialist keyworkers to support autistic people through complex care planning may be especially worthwhile. Additionally, services could consider integrating methods commonly used by disability researchers into care planning paperwork. For example, including visual verbal prompts [92], photo elicitation [93] or art-based methods [94] into care planning paperwork may help to remove barriers experienced by autistic young people with the use of standardised documentation. By integrating these approaches into the Family Plans used within the LDAP for example, the paperwork could be used as an active conversation scaffold to discuss decisions rather than a bureaucratic tool.

Future research may consider exploring how autistic young people experience shared decision-making in other contexts and examine the features of practice that were highlighted as useful in this study. In particular, the act of *enabling autistic thinking patterns* and *creating conditions for reciprocity and dialogue* simultaneously may warrant further investigation to see whether these two approaches (and the associated strategies used by the keyworkers) are helpful to other autistic young people when participating in conversations about significant decisions.

## Supplementary Information

Below is the link to the electronic supplementary material.

Supplementary Material 1: Additional Material 1 (.pdf) – Young Person Interview Schedule.

Supplementary Material 2: Additional Material 2 (.pdf) – Parent/carer Interview Schedule.

Supplementary Material 3: Additional Material 3 (.pdf) – Keyworker Focus Group Schedule.

Supplementary Material 4: Additional Material 4 (.pdf) – Deductive coding frame.

## Data Availability

Data shared in this paper are still being analysed as part of a doctoral programme and are not yet available in public archives. Additionally, due to the sensitive nature of the data and the agreements made with participants at the point of consent, supporting data cannot be made openly available. Data will be submitted to the institutional repository: further information about the data and conditions for access are available from the University of Southampton repository at [https://eprints.soton.ac.uk/] (https://eprints.soton.ac.uk/).

## Footnotes

1 By virtue of the Family Law Reform Act (1969) [15], young people in England age 16 are presumed in law to be competent to make decisions about their medical care and support. This can only be overridden in exceptional circumstances.

2 In line with the neurodiversity movement which views autism as a natural human neurodivergence [16], this paper refers to ‘autistic’ and ‘non-autistic’ people. This language choice recognises autism as a fundamental part of an autistic person’s identity and reflects the preference for ‘identity-first’ language found across many autistic communities [17].
