# Supplementary material for: ‘Bridging the gap’: exploring shared decision-making with autistic young people within an NHS Learning Disability and Autism Keyworker Programme in England
Source: BMC Health Serv Res. 2026 Feb 2;26:320. doi: 10.1186/s12913-026-14025-z (PMC12952178; doi:10.1186/s12913-026-14025-z)
Supplement: Supplementary file 1 — Supplementary Material 1: Additional Material 1 (.pdf) – Young Person Interview Schedule. [file 12913_2026_14025_MOESM1_ESM.pdf]

Young Person Interview Schedule

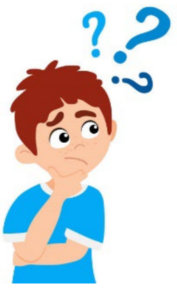	1. What does shared decision-making mean to you?
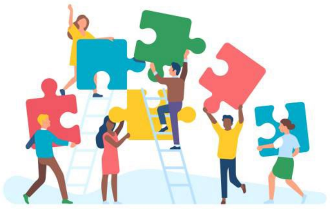	2. What has been your experience of shared decision-making with your keyworker?
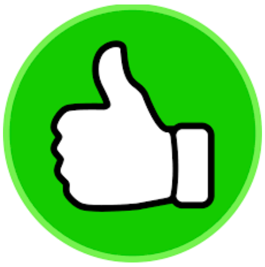	3. When have you felt included within decision-making?<br><br>Can you give me some examples of what helped you to participate?
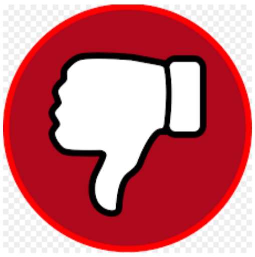	4. When have you not felt included within decision-making?<br><br>Can you give me some examples of what stopped you from being included?

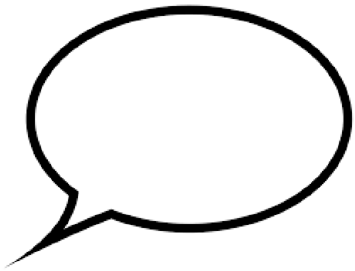	5. How does the words your keyworker uses help you or confuse you?
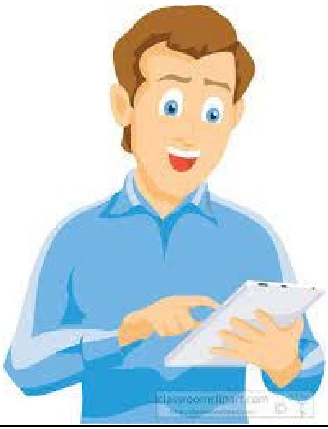	6. What information helps you to discuss decisions with professionals?
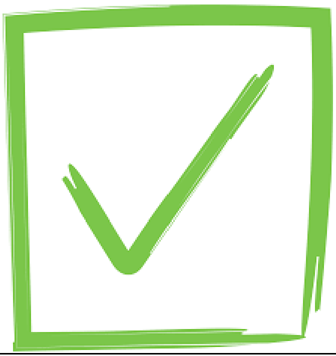	7. What helps you to be fully involved in decision-making conversations?
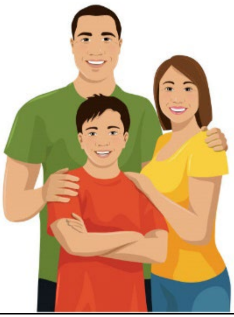	8. Do you think your voice is heard equally in decision-making conversations?
