# Supplementary material for: ‘Bridging the gap’: exploring shared decision-making with autistic young people within an NHS Learning Disability and Autism Keyworker Programme in England
Source: BMC Health Serv Res. 2026 Feb 2;26:320. doi: 10.1186/s12913-026-14025-z (PMC12952178; doi:10.1186/s12913-026-14025-z)
Supplement: Supplementary file 2 — Supplementary Material 2: Additional Material 2 (.pdf) – Parent/carer Interview Schedule. [file 12913_2026_14025_MOESM2_ESM.pdf]

Parent/Carer Interview Schedule

1. What does shared decision-making mean to you?

2. What has been your experience of shared decision-making with the LDAP?

3. When have you felt your child has been included within decision-making? Can you give me some examples of what enabled them to participate?

4. When have you felt your child was not included within decision-making? Can you give me some examples of what prevented them from being included?

5. How does the language professionals use impact your child's involvement in decision-making conversations?

6. How do professional attitudes impact your child's involvement in decision-making conversations?

7. What helps your child to be fully involved in decision-making conversations?

8. Do you think your child's voice is heard equally within decision-making conversations about their care and support?
