# Supplementary material for: ‘Bridging the gap’: exploring shared decision-making with autistic young people within an NHS Learning Disability and Autism Keyworker Programme in England
Source: BMC Health Serv Res. 2026 Feb 2;26:320. doi: 10.1186/s12913-026-14025-z (PMC12952178; doi:10.1186/s12913-026-14025-z)
Supplement: Supplementary file 3 — Supplementary Material 3: Additional Material 3 (.pdf) – Keyworker Focus Group Schedule. [file 12913_2026_14025_MOESM3_ESM.pdf]

Keyworker Focus Group Schedule

1. What does shared decision-making mean to you?

2. How is shared decision-making enacted with the autistic young people you support?

3. Can you give me some examples of when you feel shared decision-making has been really successful? What made it successful?

4. Can you give me some examples of when you think shared decision-making has not been successful? What made it unsuccessful?

5. How do autistic young people express agency in shared decision-making from your experience?

6. Do you think autistic young people’s voices are heard equally within decision-making conversations?

7. What do you think helps autistic young people participate in shared decision-making conversations?
