# Supplementary material for: ‘Bridging the gap’: exploring shared decision-making with autistic young people within an NHS Learning Disability and Autism Keyworker Programme in England
Source: BMC Health Serv Res. 2026 Feb 2;26:320. doi: 10.1186/s12913-026-14025-z (PMC12952178; doi:10.1186/s12913-026-14025-z)
Supplement: Supplementary file 4 — Supplementary Material 4: Additional Material 4 (.pdf) – Deductive coding frame. [file 12913_2026_14025_MOESM4_ESM.pdf]

Deductive coding frame reflecting Bakhtinian concepts on dialogue

Bakhtinian Concept	Code Label	Code Definition	What does this mean?	What might this look like?
**Otherness**	**Open to change**	Willing to consider new ideas or perspectives	Someone demonstrates the ability and willingness to consider another's perspectives and to be empathic towards their position and view. They are willing to adapt and change their view based on the experiences and views of another.	Someone enters an exchange with an 'open mind'. Their view or perspective may be changed following an exchange with another person. Two people are responsive to each other's words and actions. For example, a conversation follows natural twists and turns as each person is guided and influenced by what the other says. A contraindication of this is when someone shuts down another person's perspective, dominates communication or seems unwilling to consider someone else's views.
	**Understanding is co-created**	Meaning is shaped and generated through interaction with another. There is a recognition of a duality inherent in developing understanding.	Words do not convey a neutral and objective meaning. Meaning within dialogue is therefore co-constructed as both communication partners take responsibility for developing a shared understanding. Meaning and understanding is negotiated reciprocally, as each person influences the other.	People may go back and forward within an exchange to try and negotiate meaning together. In practice, person A reflects on the perspectives of person B and integrates those views into forming their own. For example, having listened to the way person A explains their view on X, person B may alter their view on X. Within a conversation, someone may check their understanding of another person's explanation – "Can I check, do you mean …? / "What do you mean by that?" Or they may check how another person has interpreted their own speech – "What does that mean to you?" "Does what I've said make sense to you?" People are reflective of how what they say or do is being interpreted by another person. A contraindication of this is when people misunderstand each other or assume meaning without checking.

**Polyphony**	**Multiple authentic voices**	The authentic voices of multiple people are evident within a dialogue. Each voice is representative of its own beliefs and world view.	Multi-voicedness is at the heart of dialogue. It means that multiple unique perspectives are shared within a communicative space.	Multiple authentic voices are evident when people share their own views and perspectives within a social exchange. For example, during a meeting, each member brings their own outlook to the discussion of a shared topic, rather than simply repeating the views of the manager. This may be rooted within practical experience – "when I tried this, I found that…" or it may be rooted in opinion – "I think this may be useful because…"
	**Single voice**	There is no dialogue. Or multiple people are talking. However, their authentic voice is not represented.	Just because multiple people are speaking, this does not mean they are all representing their individual world view. People can act as mouthpieces for external narratives – speaking in line with other person/entities' views, rather than their own. When single voice is exhibited, there is a drive towards a single and unified narrative. Diverse voices may be shut down or silenced.	Single voice is evident when a single perspective is advanced even though multiple people are talking. This can occur in a range of social circumstances. For example, during a meeting at work a manager may ask the rest of the team if they agree with the planned action. Team members may superficially agree and share the managerial narrative; despite admitting they actually disagree with the proposed approach after the meeting has ended. Single voice may also be occurring when a person's voice is assumed or misattributed. For example, someone may interpret someone's silence in response to a question as agreement, but this is not their true position. The conversation has therefore only represented a single voice. Similarly, someone's voice may be shut down or silenced directly by not asking for their view or perspective, or it may be silenced indirectly by using sociolinguistic tools to supress their voice. For example, providing someone with a form to share their views that they are unable to read, access or complete. Or by using dismissive language that closes down dialogue such as "this is just the way it is", or "you probably won't understand this" or "you don't need to know that".

**Heteroglossia**	**Unifying language**	Language is drawn towards a unified and dominant linguistic identity.	There are always competing forces acting on language. Bakhtin suggested that a centripetal force represents the dominant force trying to unify language and create homogeneity. Dominance is often connected to hierarchy as the more authoritative language becomes, the more we feel its power.	The use of unifying language is underpinned by dominance. It may therefore relate to organisational hierarchy or neurotypical communication styles. For example, professionals may use 'professional jargon' rather than common language during meetings. This may include the use of acronyms or hyper-formal descriptions of common events. Terminology and language structures are expected to conform to 'company policies'. Similarly, unifying language is evident where normative use of language, grammar and syntax are considered superior and non-negotiable. For example, speaking with a relatively consistent pace and using varying intonation.
	**Individualising language**	Language is pushed away from centralised uniformity and towards individualisation.	There is an alternate force known as a centrifugal force acting against unifying language. This represents an individuality that rejects dominance and displays uniqueness. This force can be considered destabilising, as the form or use of individualising language may be unconventional.	Centrifugal force is evident in all language that opposes structural and linguistic 'norms'. For example, the use of slang or uncommon phrases within a professional context. It may also be seen in non-dominant uses of language patterns. For example, unusual levels of repetition, elongation of words or heavy stress/emphasis on particular words.
**Carnival**	**Carnival**	Language and/or action that disrupts the status quo. Language and/or action that	Carnival acts and language can challenge power structures and social expectations. There is an air of disrespect for authority	Acts that seem to depart from social norms can be considered carnivalesque. In spoken form, this may include a refusal to participate in a conversation by not speaking at all, using expletives to highlight frustration, or unusual levels of repetition. Actions may include sitting on the floor, informal or unusual

		represents an unusual social act.	within carnivalesque acts, as language and behaviours undermine and mock dominant social norms.	dress/hair etc., walking out of meetings, or deliberate pen tapping during a time of silence. Laughter is common within carnival.
